# Perinatal mental health equity: patient perspectives on virtual collaborative care providing what women need now in the post-COVID era

**DOI:** 10.3389/fpsyt.2026.1858606

**Published:** 2026-09-16

**Authors:** Angela Marie Johnson, Rena Menke, Olivia Yang, Jordan Charles, Cecilia Martinez-Torteya, Kari Foote-Johnson, Maria Muzik

**Affiliations:** 1 Zero to Thrive, Department of Psychiatry, University of Michigan-Michigan Medicine, Ann Arbor, MI, United States; 2 Program for Multicultural Health, Department of Community Health Services, University of Michigan Health, Michigan Medicine, Ann Arbor, MI, United States; 3 Department of Obstetrics and Gynecology, University of Michigan-Michigan Medicine, Ann Arbor, MI, United States

**Keywords:** collaborative care model, COVID19, intervention, perinatal care, virtual mental health

## Abstract

**Introduction:**

The COVID-19 pandemic increased perinatal mental health needs and disrupted routine obstetric care, disproportionately affecting Black and economically disadvantaged communities. Michigan’s Perinatal Collaborative Care Model (Peri CoCM), a publicly funded virtual program, launched during the pandemic and continued after the public health emergency. This qualitative study examined participant experiences with Peri CoCM, including perceived usefulness, acceptability, and barriers/facilitators to engagement, particularly among marginalized perinatal people to inform post-COVID service delivery.

**Methods:**

Eligible participants were pregnant or up to one year postpartum Peri CoCM patients recruited from a May–July 2025 referral cohort. Nine women ages 27–42 completed one-hour, semi-structured Zoom interviews between August and September 2025; the sample intentionally overrepresented self-identified Black women. Interviews were recorded, transcribed, verified against audio, and analyzed by a coding team using grounded-theory–informed thematic analysis with constant comparison.

**Results:**

Participants reported positive experiences with Peri CoCM across four thematic areas: (1) Pathways to care, (2) Perceived impacts of program, (3) Importance of culturally responsive care, and (4) Recommendations for program optimization. Trusted referrals from obstetric and other providers facilitated entry into care, often for anxiety, depression, grief, or first-time mental health support. Participants described Peri CoCM as accessible, supportive, and valuable during pregnancy and postpartum. Virtual options, flexible scheduling, compassionate behavioral health clinicians, and consistent follow-up reduced barriers and promoted openness. The short-term model helped some transition to longer-term therapy, while clinicians supported insurance navigation, medication decisions, and community resource connections. Participants gained coping skills, validation, and self-understanding. They emphasized culturally responsive provider fit and recommended longer continuity, broader wraparound services, and sustained no-cost access to reduce stress and improve engagement.

**Conclusion:**

Michigan’s Peri CoCM was perceived as a low-barrier, relationship-centered, culturally responsive virtual pathway supporting perinatal wellbeing and systems navigation in the post-COVID era. Cost transparency and flexible delivery were central to acceptability and expanded access to equitable care.

## 1 Introduction

March 2020 marked the start of the COVID-19 pandemic (1), and the rapid emergence of intertwined physical (2) and mental health (3) challenges for pregnant and postpartum people. Moderate-to-severe COVID-19 infection during pregnancy increased the risk for serious maternal morbidity (e.g., preeclampsia, preterm labor) and mortality (2). These risks unfolded in the context of longstanding racial inequities in U.S. maternal health; during the pandemic, maternal mortality rose sharply for Black and Hispanic women from 68.0 and 28 deaths per 100,000 live births, respectively, compared to 26.3 for White women (2) widening preexisting disparities (4). In parallel, pandemic-related social adversities (e.g., work-related viral exposure, economic strain, reduced social interaction, and constrained social and childcare supports) disproportionately affected Black communities (5, 6), compounding stress during pregnancy and postpartum.

At the same time, access to routine obstetric and perinatal support was disrupted by canceled or delayed visits, shifts to remote care, and infection-related concerns (7). These disruptions reduced opportunities for screening and timely response to psychological and psychosocial needs creating inequities in care as individuals’ needs were not identified and addressed. Consistent with these conditions, rates of perinatal depression and anxiety increased substantially from an estimated 11–17% pre-COVID-19 to approximately 36% during the pandemic (8), with evidence of an even higher burden among Black perinatal people (9). Although the World Health Organization declared an end to the COVID-19 public health emergency in May 2023 (10), many stressors and access barriers persisted, and Black perinatal people have continued to experience disproportionate perinatal mental health burden in the post-COVID era (11).

Contributing factors include compounded stressors (e.g., childcare, food insecurity, return-to-work pressures, financial instability) (12), reduced or delayed prenatal care and fewer in-person supports and restricted access to community-based resources such as doulas (13, 14). For many Black women, these structural barriers intersect with experiences of culturally unresponsive care, perceived trauma, dismissive communication, and racism or bias within perinatal healthcare settings, all dynamics associated with mistrust and underutilization of needed services (15–18), all of which may have been preexisting but were compounded during COVID.

Together, these conditions underscore an ongoing need for accessible, respectful, and culturally responsive perinatal mental health care pathways that can function during periods of health system disruption and beyond. In response to the pandemic-era access crisis, the Michigan Perinatal Collaborative Care (Peri CoCM) model was launched in 2021 and has continued operating post-pandemic, serving hundreds of diverse perinatal women and families. Given continued inequities and unmet needs, it is critical to evaluate whether such models deliver low-barrier support, particularly for Black women and to identify barriers, facilitators, and lessons learned that can strengthen access and effectiveness in the post-COVID era (19, 20).

### 1.1 Michigan’s perinatal collaborative care model

Michigan’s Perinatal Collaborative Care Model (Peri CoCM) was developed to create new, accessible pathways to perinatal mental health care and has been implemented statewide with public funding (21). Building on the evidence-based collaborative care approach where an obstetric team partners with a behavioral health care manager and a psychiatric consultant to track patients, deliver brief psychotherapy, and optimize medication management for perinatal mood and anxiety disorders (PMADs) (22), Michigan’s Peri CoCM was adapted for greater flexibility, accessibility, cultural responsiveness, and equity during the COVID-19 crisis and beyond.

Michigan’s Peri CoCM is embedded within the Michigan Clinical Consultation and Care (MC3) program, the state’s Psychiatry Access Program launched in 2012 (23). In 2020, MC3 added a perinatal CoCM arm that centralized behavioral health clinicians (BHCs) within the MC3 team and delivered services virtually (phone/video/text/email), rather than embedding staff in individual health systems. Care is free and available statewide to pregnant and postpartum patients (up to one year postpartum), regardless of health-system affiliation, using patient-preferred timing and communication modality. Key components include outreach within 24 hours of referral (self or provider), validated assessment, individualized care planning that incorporates social and mental health concerns, symptom monitoring, weekly psychiatric consultation and coordination with obstetric providers, brief evidence-based psychotherapy, referral to higher levels of care when needed, and follow-up through one year postpartum.

Delivered by master’s-level licensed clinicians with perinatal and infant mental health training, the program emphasizes patient-centered, relational, and culturally responsive engagement. Its design is intended to reduce common barriers to perinatal mental health care such as limited detection, poor access and continuity of care, and negative healthcare interactions (24, 25) through rapid “warm” outreach, motivational and culturally responsive communication, flexible telehealth delivery, and tangible resource support (26, 27). The model demonstrates equitable care by identifying individuals’ needs through the evaluation process and then providing the needed services to support that person’s health ensuring equitable outcomes. Culturally responsive care involved recognizing participants’ coping strategies, building on strengths, understanding their reasons for seeking services, and providing respectful, nonjudgmental support in therapy (28). While the program was established during the height of the COVID-19 pandemic, the program is ongoing and continues to serve perinatal women now in the post-COVID-era.

This qualitative study examined participants’ experiences with Michigan’s Peri CoCM in the post-COVID era, focusing on perceived usefulness, acceptability, and barriers and facilitators to engagement. Rather than evaluating clinical outcomes or program effectiveness, the study centered participants’ accounts of how they experienced the program, what they valued, and how it addressed their perinatal mental health and support needs. Because Peri CoCM was developed during the pandemic and continued afterward, it offers an opportunity to understand experiences with a virtual, publicly funded perinatal mental health care model, particularly among Black women disproportionately burdened post-COVID. Findings can inform ongoing refinement of the model and support efforts to provide equitable, low-barrier access to perinatal mental health care.

## 2 Methods

### 2.1 Study design and participants

Women receiving services in the Michigan Peri CoCM were eligible to participate in this qualitative program evaluation. Peri CoCM patients are 18 years or older, pregnant or up to 1 year postpartum, racially and socioeconomically diverse with both private or public (Medicaid) insurance coverage and referred for or reporting perinatal mood and anxiety concerns, but without active suicidality requiring a higher level of care. Since program inception in 2020, the program served annually an average of 310 women, (range: 65-557) for a total of 1563. For demographics of Peri CoCM participants, see **Table 1**.

**Table 1:** Demographics and program engagement contrasting study participants (N = 9), referral sample (N = 183), and total Peri CoCM population served between 2021 to 2025 (N = 1563).

Variable	Study participants (N = 9) n (%)	Referral sample (N = 183) n (%)*	Peri CoCM participants (N = 1563) n (%)
Age	Mean = 32.9 Range = 28 to 42	Mean= 31.6 Range= 19 to 44	Mean = 30.1 Range = 15 to 48
Race			
*White*	4 (44.4)	114 (62.30)	894 (60.0)
*Black*	5 (55.5)	49 (26.78)	471 (32.6)
Insurance type*			
*Private*	5 (55.5)	88 (48.09)	674 (48.9)
*Public*	3 (33.3)	66 (36.07)	680 (49.4)
*None*	1 (11.1)	1 (0.55)	24 (1.7)
Pregnancy status			
*Pregnant*	6 (66.6)	89 (48.63)	697 (45.8)
*Postpartum*	2 (22.2)	87 (47.54)	762 (50.0)
*Other*	1 (11.1)	6 (3.28)	65 (4.3)
Program engagement			
*BHC Encounters*	Mean = 3.1 Range = 1 to 6 44% 1 to 2 encounters 34% 3 to 4 encounters 22% 5 to 6 encounters	Mean = 4.8 Range = 1 to 19	Mean = 6.3 Range = 1 to 38**

*Missing information for some participants.

** Funder (State of Michigan) restricted in mid-2025 the number of encounters allowable to < 6 and recommended for services >6 utilization of insurance coverage (telehealth).

We recruited for the qualitative substudy during a 3-month window (May to July 2025) from the Peri CoCM participant pool (total *N* = 183); invitations to a qualitative interview were provided via phone call (or voice message), texting or email. The invited sample closely matched overall referral population demographics and was predominantly White (64.02%), followed by Black (27.44%). Smaller proportions of participants identified as Hispanic (8.54%), Other race (2.44%), Native/Indigenous (1.83%), South Asian (1.22%), Arab (1.22%), and Native Hawaiian or Pacific Islander (0.61%). East Asian respondents were not present in this sample (0%). During recruitment, participants were informed that they would receive a $100 gift card as a token of appreciation for their participation.

Up to five attempts were made to connect with potential participants; 151 did not respond, and 20 responded and were not interested. Of the remaining twelve respondents, three were unable to complete the interview due to time constraints. A total of nine women completed the qualitative interview; demographically active participants were: Black (5ct) and White (4ct) women, privately health-insured (5ct), publicly health-insured (3ct) and (1ct) no insurance. (see **Table 1** for characteristics of study participants and full referral pool sample characteristics, and **Figure 1** for Recruitment Flow Figure). At the time of the interview, all nine participants were actively engaged and enrolled in the program.

**Figure 1:**
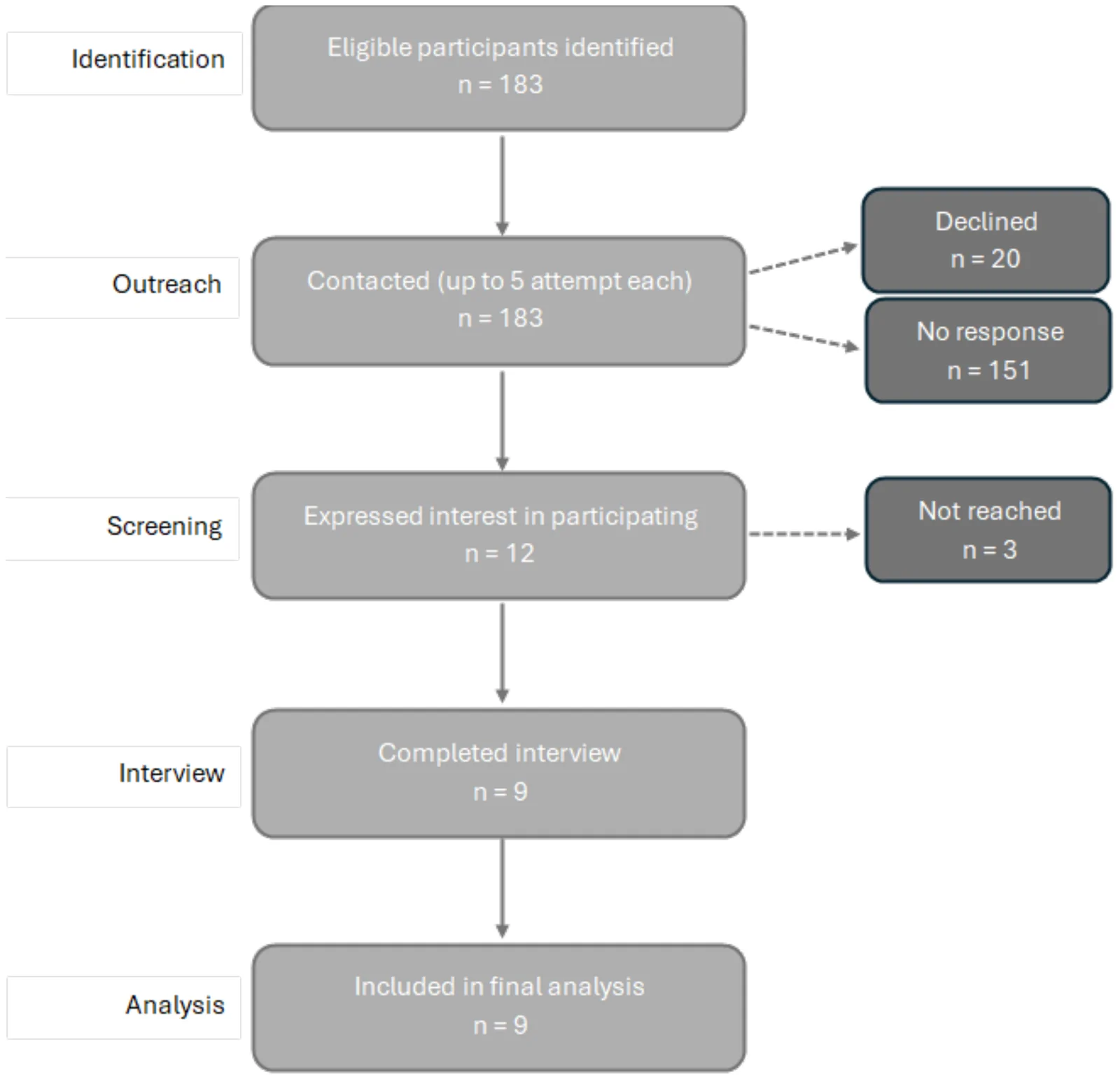
Recruitment flow chart.

Participants were recruited from the broader Peri CoCM participant pool based on referrals made during the recruitment window (May–July 2025). At the time of recruitment, individuals were at varying stages of engagement with the Peri CoCM program. The initial intent of recruitment was to prioritize individuals who were more recently enrolled in the program to capture experiences during active engagement. However, as recruitment progressed and response rates were low, sampling expanded to include participants across a broader range of program exposure, including individuals who were actively engaged in services, those with more intermittent or reduced engagement, and those who had completed or discontinued participation in the program.

Engagement level at the time of recruitment was not used as a strict eligibility criterion. While we initially attempted to purposively reach participants closer to program initiation, this was not consistently achievable due to response rates and scheduling constraints. As a result, variation in program engagement was not formally stratified or controlled during sampling. However, this variability was considered during analysis, and we remained attentive to how differences in stage of engagement may have shaped participants’ experiences and reflections on the program.

### 2.2 Data collection

Virtual one-on-one interviews were conducted in August through September 2025 via Zoom (Version 6.5.7) video call platform by study team members trained in qualitative interviewing skills Prior to the interviews, interviewers discussed with potential participants, study purpose, significance, methodology, and the expected duration of the interview. Participants were told measures to help ensure confidentiality and anonymity, and their consent obtained to record and analyze interview data. Participants were also informed that their access to Peri CoCM services would be unaffected regardless of their participation and responses in this study. To keep their identities anonymous, every participant was assigned a participant number with first and last initial of their name. Interviewees were offered $100 gift card as an incentive for participating in this study.

A semi-structured interview guide was developed to facilitate interviews (see **Table 2** interview guide). The guide was developed iteratively by a multidisciplinary team with expertise in perinatal psychiatry, behavioral health, program implementation, and evaluation. The interview domains were developed to align with the study objectives of understanding participants’ experiences with Peri CoCM, including perceived usefulness, acceptability, and barriers and facilitators to engagement. Key stakeholders reviewed draft questions for relevance, clarity, and alignment with study goals. Stakeholder feedback informed the refinement of question wording, sequencing, and domain coverage and the final interview guide was established through team consensus. The guide permitted interviewers to organize and contextualize participant feedback and to support interpretive rigor and consistency based on Codebook framing perspective (29). while still having autonomy to divert and explore pertinent ideas that may surface in the course of the interview (30). Guided by principles of patient-centered care, interviews were designed to elicit participants’ reflections of their experiences, perceived personal impact of the Peri CoCM participation, perceived value of Peri CoCM participation, barriers and facilitators to care engagement, the extent to which services aligned with their support needs and preferences, and recommendations for program improvement.

**Table 2:** Interview guide: questions and follow-up probes.

Interview questions and follow-up probes
• What motivated you to sign up for the program? • What was your impression of the program when you first heard about it? • What has kept you participating in the program? • Were there things you were hoping for that you have not experienced through the program? • Tell me about your experience receiving mental health support with program. What has it been like? • What aspects of the program did you value most? The least? • Are there aspects of the program that were attentive to your culture, background, and experiences? Were there aspects that were not? • Has this program impacted your well-being? How so? • Sometimes, people come into the program, and they may not have resources like food, insurance, or primary care providers. Did program connect you to any services or resources? If yes, what services or resources were you connected with? • How easy or difficult was it to access those services or resources in your community? • How helpful were these services/resources? If no, were there barriers to getting connected? • What were the most important ideas and skills you learned in the program? *Facilitators: first see which concepts or skills come up organically and probe for other concepts or skills.* • How did you receive services (e.g., phone, text, video chat, email)? • What were your likes and dislikes for each means of communication? • Tell us about your experience in receiving the screening questionnaires via text or email each month. What did you like? What did you dislike? • We were able to offer this service at no cost to you. What was it like to have this at no cost to you? • If this program billed insurance, would it influence your decision to participate in the program? How so? If yes and they did not mention their co-pay: With your current insurance, do you have a copay or deductible? What is it? • Does this impact your ability to access mental health care? • For our last question, we want to learn how we can make services better in the future for patients like you. I want to imagine that you have a magic wand that allows you to design perinatal care any way that you want: money, time, and resources are unlimited. How would you want to receive your care in pregnancy and after birth or during this time? It is ok to think big and outside of the box! • Is there anything about the care you received in program that you would change? • Is there any other information you would like to share that we didn’t touch on?

Participants were encouraged to take breaks as needed and to choose the best possible quiet environment free from disruptions. Throughout the interview, the interviewers listened attentively, encouraged open expression of feelings and experiences, and adjusted questioning methods as needed. Non-verbal behaviors, such as tone of voice and emotions, were also noted. Probing questions and statements were used, such as “Can you provide more detail?”, “What was that like”, “Please say more”, and “Help me understand.”

Strategies were implemented to ensure the accuracy and trustworthiness of the data and its analysis. Interviews were digitally recorded and transcribed into written format using artificial intelligence (AI) assisted Zoom real-time automated captions and post-meeting text transcripts (Version 6.5.7) and Adobe Premiere Pro software (Version 25.6). Study team members documented participants’ experiences in line with the research questions while engaging in reflexive note taking (31). Prior to thematic analyses, the coding team members listened to all digital recordings and compared the transcripts to review accuracy of AI generated transcriptions, which were subsequently corrected. No generative AI tools were used for data analysis, interpretation, or manuscript writing. Interviews lasted approximately 30 to 65 minutes each.

### 2.3 Coding and data analysis

All interviews were read, coded, and discussed by the first, third, and fifth authors, all of whom identify as women of color with no pre-existing relationship to the participants. The coding team met regularly for analytic debriefing, during which coders discussed emerging interpretations, reflexive observations, and coding decisions.

Data analysis was guided by Braun and Clarke’s (29) reflexive thematic approach whereby participants’ accounts were carefully reviewed and interpreted through six recursive phases. This method for analysis was chosen as it allows researchers to thoughtfully and collaboratively engage with and interpret data to generate themes based on their expertise, skills, and training as social scientists. Coders first read transcripts in full to familiarize themselves with respondent’s participants’ narratives and reported experiences.

Thereafter, they developed a codebook using the interview guide as a “framework map” to code different data-set segments (29). After individually coding the first few transcripts, coders convened to discuss and resolve differences in interpretation and create a consensus code framework. The framework was then deductively applied to the remaining transcript data, while new or unexpected content was assigned additional codes as needed. The full code team then discussed, developed, iteratively through multiple reviews and discussions, the final themes. Data was summarized and interpreted through these fully explored themes (32).

## 3 Results

Participants discussed in detail their personal experiences with the program, the ways they perceived and described Peri CoCM participation and impact, and recommendations to enhance care. Four major theme areas emerged: (1) *Pathways to care*, (2) *Perceived usefulness of program*, (3) *Importance of culturally responsive care*, and (4) *Recommendations for program optimization,* and are elaborated on below. **Table 3** shows a summary of the themes, subthemes, and brief descriptors.

**Table 3:** Themes, subthemes and descriptions.

Themes	Subthemes	Description
Pathways to care	Trusted referral	Positive response to referrals from trusted providers particularly those with pre-existing and/or current mental health challenges.
Perceived usefulness of program	Virtual Accessibility & Flexibility	Flexible scheduling and technology-enabled care (e.g., option of video visits and text communication) improved accessibility and supported ongoing engagement.
	Compassionate Care	Attentive, non-judgmental, and warm compassionate BHC care left women more willing to engage openly motivated them to engage
	Short term model as a bridge	Positive experiences with the short-term care model promoted self-care behaviors and increased willingness to pursue longer-term mental health treatment.
	Systems navigation support and resources	Assistance with navigating healthcare and social service systems was viewed as a major benefit. Guidance with insurance, medication decisions, and connections to other resources (e.g., breastfeeding supports, doulas, housing) were discussed.
	Learning selfcare skills	A greater sense of self-understanding and practical skills which helped women manage life difficulties.
Culturally responsive care	Participant-provider fit	Care tailored to recognize and support their unique personal, cultural, spiritual or social needs and perspectives.
Recommendations for program optimization	Continuum of Care beyond immediate postpartum	Longer-term care to follow women through pregnancy and postpartum, with check-ins and follow ups—especially when women experience difficulty of loss.
	Wraparound resources and services	Connection to more resources, services (e.g. lactation specialist, midwife, or doula), education, and community information
	Affordability	No-out of pocket cost structure, describing it as reducing stress and removing a major barrier to mental health care regardless of insurance status.

### 3.1 Pathways to care

Participants discussed how trusted provider referrals, accessibility, and scheduling convenience served as pathways to care, igniting interest and early access. Discussions revealed that participants often had pre-existing and/or current mental health difficulties, most commonly anxiety or depression, and they felt encouraged by a trusted referring healthcare provider to consider participation in the program as a “helping hand”. Several participants described this as their first experience with mental healthcare and reported participating to seek support for pre-existing or current mental health concerns during the perinatal period.

*“I was having some pretty severe anxiety about, I don’t know, it was like four months postpartum, so I wasn’t like quick to recognize that’s what it was, really. So my doctor referred me to the program.” (42 y/o, White participant)*

*“I actually spoke with my, oh, who is she, my OBGYN nurse at the time and social worker. They were mentioning different resources for me, especially with dealing with postpartum in my previous pregnancy.” (34 y/o, Black participant)*

A number of women described distressful circumstances during their pregnancy which, coupled with their trusted provider’s referral, influenced their decision to utilize the Peri CoCM program.

*“Well, we had, you know, just gotten the bad news, basically, at an ultrasound of our pregnancy. And so it was offered to me, and I didn’t even know that really existed. It was like a short-term counseling is what it was presented to me just to help support me. So that was mainly like the basis of what I was interested in, just because I never would have personally reached out to anyone myself for help. Honestly*, *I’ve just never done therapy or anything of any sort. I guess, never felt the need, but this particular situation I’ve never had to navigate before.” (30 y/o, White participant)*

*“I guess I signed up because I … have a lot of anxiety … like, I’ve had anxiety my whole life. And then being pregnant, I went through a couple … different times that I had perinatal depression, and … So I thought it was a better idea to sign up and get into it before, because I thought … I don’t know how postpartum will be, so…” (27 y/o, White participant)*

### 3.2 Perceived usefulness of program

Several elements facilitated positive perceived usefulness of the PeriCoCM and were grouped as five subthemes:

#### 3.2.1 Virtual access and flexibility

Participants in this study viewed virtual access and flexible scheduling as a major strength to the Peri CoCM program. Participants valued multiple telehealth options, including phone, text, Zoom, and email, and the ability to switch modalities as needed. Participants described these options as fitting more easily into busy lives and as reducing perceived logistical barriers to participation.

*“I actually really appreciate telehealth. I think it’s a lot more convenient than in person.” (33 y/o, White participant)*

*“I would say I like the phone a lot better, just because I didn’t have to, like. If I look at, like, I’m super pregnant, so … [I prefer] the phone over the Zoom. But it was still nice to, like, actually see the person, so she would ask me, do you want to be on … Zoom or phone, which I appreciated.” (33 y/o, Black participant)*

*“And I’m just like, not one to really usually open up about things or be like vulnerable to people. So, seeing their face [on Zoom] and like, just seeing their mannerisms as you’re talking to them was really helpful and feeling more comfortable.” (30 y/o, White participant)*

*“Texting is a wonderful thing, especially if you’re on the go. Email is very helpful to go back on the information. If you forget something, it’s always a good corresponding.*

*Zoom … because being not being able to meet in person, I don’t drive, so to be able to have a Zoom meeting … versus having to go into an office or having to meet somewhere, that makes it difficult … that’s extremely, extremely helpful… “Just everything is, like I said, very accessible. I am a busy mama. I’m on the go a lot.” (34 y/o, Black participant)*

*“How flexible you guys are in, like, rescheduling and, like, how understanding, like, that is a great part of your program. Yes. That can’t get her shit together. Oh, no. It’s okay. Well, you know, life happens.” (42 y/o, White participant)*

#### 3.2.2 Compassionate care

Favorable care delivered by BHCs was described as attentive, non-judgmental, compassionate and left women willing to engage openly:

*“And first time talking to her, she was very calm, not judgmental. Her tone was never judgmental, and she gave a very warm opening feeling. Having that feeling, it really set the tone for me to be vulnerable and want to communicate and talk to you [sic: BHC]. I’m a very private person so to be able to do that was good.” (34 y/o, Black participant)*

*“Just really who I was partnered with, I think she was just very attentive to everything … to like, really know what I was going through instead of just treating me like another person going through the same situation, attentive to everything … I think just being able to meet with somebody and her having such good understanding and not judging what I’m saying.” (30 y/o, White participant)*

*“… she was so consistent in reaching out to me … [it] definitely made the world of difference … if she wouldn’t have followed up like I wouldn’t have, I wouldn’t have ever even, like, you know, gotten the benefit of the program.” (30 y/o, White participant)*

Empathetic care was particularly helpful when women felt overwhelmed or lacked support:

*“It’s actually helping me. I don’t see gray as much as I used to. And what I mean by that is I’m not in a dark cloud…” (28 y/o, Black participant)*

*“Being able to not only talk about my pregnancy experience, but like … my … life … before, and, like, the things I’ve dealt with beforehand, and then being able still to discuss that, and, like, why my pregnancy I might have had depression parts and stuff. And feel validated in my feelings. That, like, I wasn’t crazy, you know? I think those things were … really, like, invaluable to me. Like, it helped a lot.” (27 y/o, White participant)*

*“Just having someone to, like. Listen to me, like … It’s a lot going on. I can’t, like always go to my [family member] … so just having someone, like, listen to me … She also made me feel validated in what I was saying and feeling.” (33 y/o, Black participant)*

*“And it was extremely beneficial having her just sit there and like, listen to me face to face. Listening even if she didn’t say anything back. So, it was just like a sounding board. But yeah, just being able to like it kind of is similar as, like writing it down, you know, you get the thoughts out of your head.”(30 y/o, White participant)*

#### 3.2.3 Short term model as a bridge

Short term support served as bridge to longer-term care and was perceived as useful facilitator in overcoming internalized help-seeking hesitancy:

*“[My BHC] gave me the idea, like, of actually just doing therapy instead of short-term. That was very helpful to me because I didn’t even think about it … Because I started talking about it, finally, like how I felt and why I felt that way. And then [my BHC] was the person that said, you know, you can absolutely do long-term therapy. And that was one of the suggestions that they gave me to help. So, yeah, it was really like, I wouldn’t have even thought of that. It’s helped, like, it helped me to see that I needed to seek some help to feel better, I think, for sure.” (42 y/o, White participant)*

*“I felt good. I have been not wanting to get therapy or anything like that because I don’t want to talk to people, but now she’s made it to me, made me feel more comfortable to continue that. So once we’re done with the program, she’s linked me with some amazing therapists to continue my journey so that I am a better me.” (34 y/o, Black participant)*

#### 3.2.4 Systems navigation and resources

BHC assistance with navigating complex systems (especially insurance), medication decisions, health information, and connections to other resource people (e.g., breastfeeding supports, doulas, housing) was viewed very favorable as women in this study juggled competing life demands and limited capacity. Participants readily accepted BHC advocacy and resource navigation support:

*“One day, I wasn’t able to get in contact with my insurance. They kept giving me the runaround. So [my BHC] was my advocate for my insurance company…. So when I say she made things so much easier for me, things that I didn’t have the [time] for or I have even thought about doing on top of it.” (34 y/o, Black participant)*

*“She definitely helped me with getting, like I said, a [specialized healthcare provider]. That was huge … She also, like I said, helped with finding doula care. (34 y/o, Black participant)*

*“I was looking for, you know, some housing resources. And she (BHC) was able to provide those as well. So it’s just she sent the link for like these are things that can be helpful for you.” (32 y/o, Black participant)*

#### 3.2.5 Learning self-care skills

Participants described gaining a greater sense of self-understanding, new perspectives, and practical skills which helped them manage life difficulties such as stress, grief and other hardships:

*“[My BHC] also gave me a lot of, like, breathing techniques and, like, a lot of resources too, for, counseling. And that takes my insurance … But she gave me, like, specialized people … She really helped me.” (30 y/o, White participant)*

*“[My BHC] had helped … about, you know, where my boundaries could be, what are healthy boundaries, and all this kind of stuff … I was able to … get everything off my chest with them … So, it’s literally … it’s a night and day difference of … who I am now as a person, and … Like, it’s okay to put myself first, you know?” (27 y/o, White participant)*

*“Yeah, I love meditation. Like, after doing it, it’s just like … It’s been so much easier to … to feel at peace, even though there’s, like, so much chaos going around in the world.” (28y/o, Black participant)*

### 3.3 Importance of culturally responsive care

Women expressed interest in care tailored to recognize and support their unique personal, cultural or social needs and perspectives. Patient–provider fit, as a subtheme, surfaced numerous times and participants valued Peri CoCM as a specialized resource to find optimal fit at the intersection of pregnancy/fertility, mental health, and cultural and spiritual awareness:

*“I found a list [of therapists] from my insurance … I didn’t feel like the people on the list were, like, a good fit, because it was, like, mostly the people who were coming up were, like, marriage counselors, and it wasn’t, like, specific to, like, pregnancy or fertility. [BHC] helped to find a good fit.” (33y/o, White participant)*

*“If possible, [I would like to] get some background information about [my] provider [ahead], because I would say usually, I would prefer a provider that looks like me … If you could pick, or at least have some background of all of the providers that are listed, so even if you want to make a request, it might not be granted, but if you want to make a request for a particular one you could. We all have different needs, and not everyone understands those needs.” (33 y/o, Black participant)*

*“Religion is very key for me. In my family, and [my BHC] also being a devout Catholic, she, used some of the stuff, like give me reminders. And it helped ground me a lot. Obviously, it’s a coincidence, but like, [my BHC] being a Catholic, it helped tremendously, because my faith is a huge part of my life … It’s so nice to have a therapist who really understands me as a Catholic.” (33 y/o, Black participant)*

### 3.4 Recommendations for program optimization

Participants elaborated on program features and opportunities for improvement. Insights were based on their experiences with the program contextualized by unique conditions and circumstances of their lives and highlighted several elements they feel might enhance applicability and appeal to perinatal populations more generally:

#### 3.4.1 Continuum of care beyond immediate postpartum

Participants suggested that longer term care throughout pregnancy into the postpartum year, and even beyond in special circumstances such as loss experiences, would provide comfort:

*“I feel like for me specifically … would be being able to follow … like after something like (referring to grief) this happening to somebody, being able to have somebody that would follow them … maybe even meet with me like an appointment or something like just to check in. Even if it’s like prenatal visits or whatever the case may be, just like being able to … just having that, that person that … you see like yearly follow ups or something … And just, like, ‘how are you doing?’ Like, emotionally, you know, because I know my OB has mentioned a lot. So like somebody at the, the offices, you know, like that could just step in and know when you’re coming in and just step … having somebody like at the hospital or something … And so it’s just, you know, things like that that come up later that you don’t necessarily like, anticipate. And I think it would have just been helpful … how do you navigate [difficulties] it’s like a really complex situation.” (30 y/o, White participant)*

*“I wish your program could, like, be longer … I think it needs to be longer … Because things change throughout pregnancy, um, instead of having, like, only a set amount [of sessions], or whatever. It’s just … there’s things change while you’re pregnant, as well as postpartum…*

*Well, it’s hard, it’s hard. Like, just getting in the rhythm, and then, like, being sleep-deprived, and … Not … like, not focusing on you, like … it’s really hard, and so, like. You have mom brain going on, and so, um … You … you forget everything about yourself. So, um, like, just check-ins, like, how are you doing? Like, even if it’s just a text.” (33 y/o, Black participant)*

#### 3.4.2 Wraparound resources and services

Participants named access to integrated wrap-around resources (e.g., tangible help with household needs) and services (e.g., access to lactation consultant or doula) as critical:

*“I don’t know if it’s child care, like schools,…Just more resources, not just for the pregnancy, but we can help you to get the things for the pregnancy, but for the whole household, if you can. Like I said, if your baby or toddler needs school help, school clothes, if you must, shoes, whatever that may be.” (34 y/o Black participant)*

*“…Maybe like lactation support. I don’t know if that was offered because like I said, I didn’t get … I just feel like a lot of, like there’s a lot of important information out there. When should you contact your doctor, what medicines you can take? What things to avoid as far as food and drinks and things like that. You know, even like, you know, maybe, like the mask coaching and stuff like that, breathing push and stuff like that. Like I didn’t have access to that type of stuff to help me in labor. I just feel like I found myself googling a lot. Because I didn’t I didn’t know. I didn’t know what to expect. I didn’t know what was normal. How would certain things feel and none of that … You know, you being a first time mom, this is normal stuff like that.” (32 y/o, Black participant)*

#### 3.4.3 Affordability

The Peri CoCM program was provided at no cost to participants. Women, regardless of insurance status, overwhelmingly valued the program’s no-out of pocket cost structure as reducing stress and removing a major barrier to accessing mental health care:

*“It (cost) was something that I didn’t have to worry about … because in the sense of preparing for a baby, you think about your finances and stuff and, like, everything that you need to prepare for. So it was nice to find something that, you know, didn’t have to come out of pocket for.” (37 y/o, Black participant with private insurance)*

*“It was, you know, specifically mentioned that it was free for me. I think with having to go through insurance, a lot of people don’t know their co-pays or out-of-pocket costs for things like that. And so, that just seems like a lot to have to call your insurance and find out and everything like that and find somewhere that it’s going to be, like, cheaper for you or whatever the case is. So, knowing that ahead of time that it was free to me, it was less stressful and, just easier because I didn’t have to go through that whole process. You can get hit with a bill to earn like a high bill or have to pay out of pocket for, co-pays and things like that.” (30 y/o, White participant)*

*“I lost my job, and then I had COBRA. And I was losing COBRA, and like … then I didn’t qualify for Medicaid. Then I qualified for Medicaid, and it was just a lot. There was a point in time, I didn’t even have health insurance. And so, like, having this program was so helpful. And not have to worry about, oh, is this getting paid? Like, because I got, like, other things to worry about.” (33 y/o, Black participant with no insurance)*

*“I think a lot of people get like, unexpected bills after, even I’ve even called my insurance before and ask them, like my benefits or, you know, they’ll say like, this is what it would cost, but then you get a bill afterwards for something different. And so, I think just the stress of like, wondering, am I going to get a bill for this … it wouldn’t have been worth it to me at the beginning, because I was already on the fence. So, I think that would have made a difference (on whether she participated]. And I honestly, my honest opinion is I probably wouldn’t have done [it]. Seems like especially when you’re going through something like this, like a pregnancy loss of some sort, that just seems like another thing you kind of have to like, deal with at the time. And you just like, have no emotional capacity for that.” (30 y/o, White participant)*

*“I know my insurance, like, doesn’t cover mental health stuff, so that’s the hard thing … I’m not sure I can pay thousands of dollars in therapy out of pocket.” (27 y/o, White with private insurance)*

## 4 Discussion

This qualitative study explored women’s experiences with the Michigan Perinatal Collaborative Care (Peri CoCM) model, which was implemented during the height of the COVID-19 pandemic to expand access to perinatal mental health care, particularly for marginalized populations. The present study aimed to examine the ongoing utility of the Peri CoCM model as a low-barrier approach to perinatal mental health care in the post-pandemic era. In 2025, we interviewed nine women who were concurrently enrolled in Peri CoCM services. Interviews with this socially and economically diverse group yielded rich, detailed insights and provided valuable information about women’s preferences and needs for perinatal mental health care after the COVID-19 pandemic.

Participant insights highlighted perceptions of program access, perceived usefulness, and opportunities to enhance Peri CoCM utilization and effectiveness. Four thematic areas emerged: pathways to care, perceived usefulness, culturally responsive care, and recommendations for program optimization. Trusted referrals facilitated entry into care, while virtual access, scheduling flexibility, compassionate support, systems navigation, resource connection, and self-care skill-building contributed to participants’ perceptions of program value. Participants also emphasized the importance of culturally responsive care and identified emotional support, practical navigation assistance, and flexible, personalized telehealth options as critical to their well-being. These findings underscore the continued need, beyond the acute phase of the COVID-19 pandemic, for low-barrier, integrated, and comprehensive perinatal mental health supports such as Peri CoCM, especially for marginalized populations (33).

Peri CoCM uses a multidisciplinary team-based approach that connects participants, their medical providers, a behavioral health consultant, and a consulting psychiatrist. This model supports patient agency, professional collaboration, and a more comprehensive understanding of participants’ needs, consistent with prior research (24). Participants described culturally responsive care that included scaffolding individualized coping strategies, attention to personal needs, strong interpersonal connection, emotional support, guidance, and concrete resource navigation (28). By centering participants’ needs, this virtually delivered collaborative care model aimed to provide more accessible, responsive, and personally meaningful support. These findings are especially important for Black birthing women, who continue to experience disproportionately poor birth outcomes and interpersonal mistreatment in health care settings (34, 35).

Participants’ accounts align with prior research describing collaborative care models as acceptable and useful in perinatal mental health contexts (36). Participants described access to care as being facilitated by warm referrals, proactive outreach, resource navigation, and no-cost services to the participants. Together with core components such as psychiatric consultation, these features suggest the value of an integrated behavioral health model tailored to the needs of perinatal patients.

In terms of modality, participants described the program’s flexible, telehealth-enabled communication (text/phone/Zoom/email), easy scheduling and rescheduling, as highly valued features of participation. Focus on intensive outreach and flexibility in modes and times of communication, which are not always features of telehealth services, emerged as particularly important in the post-COVID era. The pandemic and its aftermath represent a period of societal loss and uncertainty. Specifically, perinatal people, and in particular Black individuals experienced increased rates of mental health difficulties exacerbated by systemic social, environmental, and other challenges that persist until now (5, 6, 8, 12, 37, 38). And, although the use of telehealth services to address psychiatric symptoms increased at the onset of, during and after the pandemic (27), those with lower incomes and education, unemployed, or living in rural areas were not as likely to access care via video (39). Our participants described that flexibility in scheduling was key given the frequency of unexpected circumstances during the perinatal period, while the BHC’s persistence in contacting them was perceived by participants as supportive of engagement, despite experiencing perinatal stressors.

When considering facilitators of the program, women described Peri CoCM as a low-barrier source of perinatal mental health support that they often entered through trusted clinician referral. Participants were referred themselves or were referred by their medical provider, and participants described staying in the program because of strong connections with behavioral health clinicians. This experience resulted in participants perceiving the program as “opening the door” to support and first-time therapy experiences. Participants described feeling inspired to continue care because they felt “seen and heard” and appreciated an open line of communication shaped by positive interpersonal, nonjudgemental connections. We may also consider that BHCs’ expertise, level of knowledge, skill sets and welcoming, reflective stance may have contributed to perceived fit and engagement. Our team was comprised of perinatal mental health experts, infant mental health-endorsed clinicians, and certified advanced drug and alcohol counselors (CAADC) with many years of practice and experience. Cultural elements were also perceived as supportive to engagement in the program, including preference for providers who shared racial/ethnic, religious, and other cultural characteristics. Understanding the factors that promote initiation and continued engagement in services is key given the high attrition rates reported by traditional perinatal mental health interventions (40).

Overwhelmingly, participants noted the positive impact of the program, perceiving improved mood/anxiety, a clearer understanding of their own needs, utilization of self-care and practical coping strategies to address the acute stressors faced during the perinatal period. Participants emphasized the benefit of the emotional and practical support, emotional safety and validation they received from the BHCs, as well as the usefulness of targeting concrete issues in their lives, such as grief support or coaching for setting boundaries. Women identified that the program’s utility was also in its function as a bridge to longer term care, as well as assistance with systems navigation (e.g., insurance, medication, referrals, breastfeeding resources, doula/housing supports), reflecting the importance of attending holistically to perinatal people’s needs, as opposed to limited focus to psychiatric symptoms.

Navigation support was an essential role and likely novel element in Peri CoCM primarily because resources are often not available to those seeking them. Human service programming and resources, including those for perinatal populations often have quick turnover in terms of active operation and services provided. It is common that they are being introduced, defunded, and reintroduced under varied capabilities quickly (41–43). This is critical as patients who participated were referred during a most vulnerable time and typically were unable to dedicate additional time to locating resources amid the complexity of changing access and availability. Women were also in a transitional period (e.g., being a first-time mother, or attending to multiple children, or having experienced perinatal loss), diminishing their emotional and logistical capacity to navigate the complex resource landscape. BHC served as trained individuals, whose primary role was to be experts in the existing resources available, and align the services available with the needs of the patient. This was achieved because of BHCs’ professional skill set and know-how in communal, regional and statewide maternal mental health care options and resources aiding in efficient care and resource navigation.

Participants reported cost transparency and the absence of unexpected financial costs as a critical factor for enhancing engagement in program participation and reducing day-to-day life stressors. The no-cost to patient structure was repeatedly framed as essential for access as it reduced stress and rumination about coverage, deductibles, or surprise bills and made engagement feasible during financially and emotionally strained periods. This underscores the importance of financial barriers in mental health service uptake and suggests the ongoing need for clear transparency in financial costs of programs. Unaffordable cost of care and inadequate healthcare insurance coverage may hinder women from utilizing mental healthcare at this crucial time in their lives (44).

In summary, this study examined whether the Peri CoCM model, which we developed during the height of the COVID-19 pandemic to address pandemic-related barriers to care, continues to offer value for perinatal women in the post-pandemic context. Specifically, we sought to identify which model features remain most meaningful to participants and which aspects of perinatal mental health care are broadly desired by perinatal women.

Findings from this study offer lessons that extend beyond crisis-driven COVID-19 care delivery and remain relevant in the current post-pandemic era. Perinatal women, particularly marginalized and Black women, continue to experience adverse mental health effects related to the pandemic and its aftermath (33). Through participants’ voices, this study highlights key elements of care that should be considered integral to perinatal mental health programs and systems. Consistent with prior literature, participants emphasized the importance of providers’ clinical expertise and cultural responsiveness in shaping perceived fit, trust, and affirming care (45). Engagement with patient-centered mental health providers, often for the first time, also appeared to shift participants’ perceptions of mental health treatment, helping to counter racially and culturally informed concerns about mental health care and care systems (24). Many participants described greater openness to continuing care after the short-term Peri CoCM intervention ended. Several additional program features emerged also as especially valued, including an easily accessible, multimodal, virtual, and no-cost care; rapid outreach; a safe and flexible first encounter; and support for ongoing engagement. Participants also highlighted the importance of expert, up-to-date resource and referral navigation, particularly for those seeking longer-term care. These findings align with prior reports showing increased engagement with virtual care models (27) and reinforce evidence that difficulty navigating resources remains a significant barrier to accessing mental health care (24).

### 4.1 Strengths and limitations

The study’s strengths include in-depth qualitative data capturing rich patient-centered perspectives on a publicly funded perinatal collaborative care model that began within and then continued beyond the COVID-19 public health crisis. Authors acknowledge that there are several limitations.

First, the study may be affected by self-selection and recall bias. It was not designed to assess effectiveness or symptom change, and the small sample size precluded formal subgroup comparisons. Second, responses may have been positively biased due to interview wording or perceived expectations. Interviewers attempted to minimize this by using open-ended questions, neutral prompts, and assurances of anonymity and confidentiality; however, interviewer bias may still have influenced responses. Third, participants with favorable experiences may have been more likely to participate, while those who were disengaged or dissatisfied may be underrepresented. Fourth, the study may also miss perspectives from individuals facing barriers such as housing instability, severe mental illness, limited digital access, or caregiving demands. A larger study sample to perform sub analyses by mental health and other potential confounders would have been desired. Fifth, the nine participants had fewer BHC encounters on average than the broader 2025 referral sample, which may have shaped their perspectives and the findings. Finally, given the small sample size, the findings are not broadly generalizable and have limited transferability. Nevertheless, we believe, the findings offer important considerations and potential strategies for future program development, if thoughtfully applied to other populations, contexts, and circumstances.

## 5 Conclusion

In conclusion, findings from this qualitative study suggest that the Michigan PeriCoCM model remains a relevant and acceptable approach to addressing perinatal mental health needs in the post-pandemic context. Participants characterized Peri CoCM as a low-barrier, flexible, and relationally supportive model of care that facilitated entry into services, promoted engagement, and addressed both emotional and practical needs during the perinatal period. Core elements perceived as particularly meaningful included trusted referrals, proactive outreach, multimodal telehealth access, scheduling flexibility, culturally responsive and nonjudgmental clinical relationships, no-cost participation, and individualized resource navigation. These findings underscore the importance of perinatal mental health models that extend beyond symptom-focused intervention to incorporate systems navigation, attention to social needs, and culturally responsive support, particularly for marginalized and Black birthing populations. Although limited by a small, self-selected sample, the study offers important patient-centered insights to inform future development, adaptation, and evaluation of integrated perinatal behavioral health programs. Continued investment in collaborative, accessible, and culturally attuned models such as Peri CoCM may be critical to reducing persistent gaps in perinatal mental health care and improving support for perinatal individuals and families.

## Data Availability

The datasets generated and analyzed during the current study are not publicly available as they contain sensitive qualitative interview data from a small participant sample, which could compromise participant confidentiality. De-identified data may be made available from the corresponding author upon reasonable request and subject to institutional review and applicable ethical and privacy requirements.
